# Effect of radiotherapy on the gut microbiome in pediatric cancer patients: a pilot study

**DOI:** 10.7717/peerj.7683

**Published:** 2019-09-23

**Authors:** Nourhan Sahly, Ahmed Moustafa, Mohamed Zaghloul, Tamer Z. Salem

**Affiliations:** 1 Biomedical Sciences Program, University of Science and Technology, Zewail City of Science and Technology, Giza, Egypt; 2 Biotechnology Graduate Program, American University in Cairo, New Cairo, Egypt; 3 Department of Biology, American University in Cairo, New Cairo, Egypt; 4 Radiation Oncology Department, National Cancer Institute, Cairo, Egypt; 5 Children’s Cancer Hospital Egypt 57357 (CCHE 57357), Cairo, Egypt; 6 Microbial Genetics Department, AGERI, ARC, Giza, Egypt

**Keywords:** Chemoradiotherapy, Response to treatment, Pediatric cancer, 16S rRNA, Rhabdomyosarcoma, Radiotherapy, Mutations, Gut microbiome

## Abstract

The incidence of pediatric cancer is lower than that of adult cancer worldwide. However, the former has detrimental side effects on the health of individuals, even after the cancer is cured, due to the impact of treatment on development. Recently, correlations have been made between the gut microbiome and cancer in several studies but only on adult participants. There is always a complication of dealing with pediatric cancer treatment protocols because they usually include a combination of chemotherapy, radiotherapy, and intensive prophylactic antibiotics. In the current study, a pilot study was conducted to analyze ten fecal samples from three pediatric cancer patients, suffering from rhabdomyosarcoma near their pelvic region, and two healthy individuals. A correlation between microbial composition and response to treatment was reported, in which the responders had generally a lower microbial diversity compared to non-responders. In addition, nucleotide changes and deletions in the tested 16S rRNA sequences post radiotherapy were detected. Despite the small sample size used in the experiments due to the uncommon rhabdomyosarcoma in children, the results can help in understanding the influence of radiotherapy on the gut microbiome in pediatric cancer patients. More work with larger sample size and different cancer types need to be conducted to understand the influence of radiotherapy on gut microbiome to mitigate the deleterious impact of radiation on treated children.

## Introduction

The human body is colonized by different microbes that vary in classification, distribution, and quantity according to the body site. The gut is considered the richest in colonization in terms of classification, diversity, and amount of bacteria. Many other factors also play a role in the microbial distribution and colonization such as age, medical condition, and environment (Morelli, 2008; Neish, 2009; Sekirov et al., 2010). The set of microbes inhabiting a certain organ or tissue is known as the microbiome or collectively defined as “the microbiome cloud” (ElRakaiby et al., 2014). The gut microbiome is highly inconstant in different states of health and disease and highly affected by different factors, starting from childbirth such as age, diet, and genetic factors (Aziz, 2009; Dominguez-Bello et al., 2010; Yatsunenko et al., 2012; Odamaki et al., 2016). Microbial imbalance as a result of infection or medication side effect is known as microbial dysbiosis (Hall, Tolonen & Xavier, 2017).

The risk of getting cancer increases with age, therefore, it is always difficult to obtain samples from children due to the low incidence of cancer among them. Although there are increasing data linking gut microbiome to cancer in adults, no comparable data were obtained from children. The administration of a combined chemotherapy and radiotherapy treatment protocol was found to be effective in improving the cancer patients’ survival rates in children (Boring et al., 1994; Meirow & Nugent, 2001). However, the treatment is usually accompanied with intensive prophylactic antibiotic courses to decrease the rate of fungal and bacterial infections (Kurt et al., 2008). Unfortunately, the side effects of such intensive treatment protocols are harmful, especially on the growing gut microbiome in infants, which makes analyzing gut microbiome in children very complicated.

Cancer patients with dysbiosis due to infection by *Escherichia coli* group 2B can lead to colorectal carcinoma (CRC) (Cuevas-Ramos et al., 2010). In addition, the variation in the microbial composition of the gut associated with adenoma increases the rate of CRC development (Shen et al., 2010). The possibility of making use of the microbial dysbiosis associated with the CRC to develop a non-invasive biomarker for early cancer detection was studied and several genes were identified to differentiate between the patients and the healthy control cohorts (Yu et al., 2017).

It is worth mentioning that even the core microbiome that is known to be constant among different people can be variable due to different factors including cancer, chemotherapy, and radiation treatment (Chase et al., 2015). It was reported that the gut microbiome can go through remolding for gynecological cancer patients undergoing abdominal and pelvic radiotherapy. The remolding was associated with diarrhea in most of the patients understudy (Nam et al., 2013). Several studies addressed the effect of chemotherapy on gut microbiome and consequent occurrence of dysbiosis. One study showed the effect of chemotherapy on pediatric cancer patients suffering from acute myeloid leukemia. It was found that during the treatment course, the microbial diversity was generally low and unstable when compared to the healthy individuals at the same age (Van Vliet et al., 2009). Another study addressed the effect of a common chemotherapy, 5-fluorouracil, on the gastrointestinal (GI) tract and reported severe damage to GI wall along with several changes in the microflora composition (Stringer et al., 2009). Moreover, it was found that germ-free mice that were exposed to lethal doses of radiation were resistant to post radiation injuries (Mclaughin et al., 1964; Crawford & Gordon, 2005). However, the effect of radiotherapy on the gut microbiome was mainly addressed in adults rather than pediatric cancer patients.

In the current study, we report the effect of an intensive and combined cancer treatment protocol on pediatric cancer patients with a minimum age of three years and half and a maximum age of seven years old. All patients involved in the study were suffering from rhabdomyosarcoma near the pelvic region. Rhabdomyosarcoma is a rare type of cancer; yet, it is highly aggressive and requires up to 50.4 Gy of gamma radiation along with a complex set of chemotherapeutic agents. It represents 3–4% of pediatric cancers and usually occurs at the age below 18 years. Due to the scarcity of the disease in children, only ten fecal samples were collected from five male participants, three cancer patients and two healthy controls. The samples were collected from patients at three different time points; before, mid (25.2 Gy–28.8 Gy), and after (50.4 Gy) radiotherapy treatment. Despite the small sample size, we inferred a relationship between the response to treatment and the microbial abundance. The possibility of radiosensitivity and possible DNA mutations in the bacterial DNA post radiotherapy were also investigated.

## Materials and Methods

### Study design and participants’ demography

Ten fecal samples were collected from three cancer patients and two healthy controls. The study included two sets of controls: self-control (same patients before starting radiotherapy sessions) and healthy individuals. Healthy is defined as not consuming any chemotherapeutic drugs or antibiotics (antibiotic-free period was defined by at least one month before sample collection) and never being exposed to radiotherapy before. All the participants in the study (patients and healthy individuals) were males with age range between 3.5 and 7 years old.

Participants in the current study were treated in Children Cancer Hospital, Egypt (CCHE 57357) and were diagnosed with rhabdomyosarcoma in the pelvic region. The treatment protocol entailed 50.4 Gy (180 cGy/fraction) that is the highest dose of radiation prescribed to pediatric cancer patients. The sample collection points were defined as follows: pre-point, before starting radiotherapy sessions; mid-point, ranging from day 12 to day 16; and the last collection point, day 26–28. All patients were treated with a complex treatment protocol including chemotherapy (Cyclophosphamide, Vincristine and Dactinomycin) and radiotherapy dose of 50.4 Gy on 28 fractions (180 cGy/fraction). The patients started the radiotherapy sessions either after 12 weeks of chemotherapy (Patient-1 and patient-3) or after four weeks of chemotherapy (patient-2). During the chemotherapy, a set of antibiotics were prescribed according to the patients’ case and needs (Table 1).

**Table 1:** Study participants.

Participant^a^	Sex	Age (Y/M)	No. of Samples	Radiotherapy fraction^b^ (Gy) and day count		Chemotherapy^c^	Antibiotics
C1	Male	3.6	1	None		None	None
C2	Male	4.5	1	None		None	None
P1	Male	7	2	0	Day 0 **(B)**	VAC	None
				21.6	Day 12 **(M)**	VC	Sulfamethoxazole and trimethoprim
P2	Male	7	3	0	Day 0 **(B)**	VAC	None
								25.2	Day 14 **(M)**	VC	None
				46.8	Day 26 **(L)**	VC	Sulfamethoxazole and trimethoprim
P3	Male	4	3	0	Day 0**(B)**	VAC	None
								28.8	Day 16 **(M)**	VC	Levofloxacin
				48.6	Day 27 **(L)**	VC	Ceftriaxone, sulfamethoxazole and trimethoprim

**Notes.**

a C, Control; P, Patient

b B, Before; M, Mid; L, Last

c V, Vincristine; A, Dactinomycin; C, Cyclophosphamide

The institutional review board approvals were granted before sample collection, from the American University in Cairo and Children Cancer Hospital Egypt (CCHE 57357). The participants have signed a child assent form and the guardians have approved the participation in study and signed a parental permission form. Fecal samples were collected from the participants with the help of their parents in sterile falcon tubes, then transferred on ice before storing in −80 °C freezer until DNA extraction.

### DNA extraction

Microbial DNA was extracted from the collected fecal samples by DNA extraction kit from stool, QIAamp DNA stool Mini Kit (Qiagen, USA), according to the manufacturer’s instruction. The protocol used enhances the non-human DNA over the human DNA extracted from the sample, through optimization of the lysis conditions as described in the manufacturer’s protocol. The DNA was eluted in 50 µl elution buffer provided by the kit and stored in −20 °C until sequencing.

### 16S rRNA sequencing

The extracted DNA was sent to Eurofins Genomics in Germany for sequencing of 16S rRNA. The sequence was performed on Illumina MiSeq platform, targeting V3–V5 variable regions of the 16S rRNA. The run was performed on 2 × 300 paired-end reads. The target region (V3–V5) length is approximately 700 bp. The obtained raw data are available on NCBI BioProject number: PRJNA545788.

### Data analysis

The 16S rRNA sequencing data received from Eurofins Genomics were already demultiplexed. The analysis was completed on QIIME 2 pipeline (version 2017.12) (Bolyen et al., 2018), q2cli command line interface. The denoise command was used followed by length trimming to 230 bp. Feature table was constructed on the same program using Deblur approach. The Deblur approach uses an error profile that operates on per-sample bases and depends on the read length and diversity in amplicon sequences. Thus, it offers a higher sensitivity and requires lower computational power compared to other OTU clustering algorithms (Amir et al., 2017). Taxonomy classes were assigned using the constructed feature table in comparison with SILVA database (Silva-119 99% OTUs full-length sequences). Unrooted phylogenetic tree was also constructed on QIIME 2 (qiime phylogeny fasttree). The taxonomic classification was appended to the feature table and exported as a biom format file. The phylogenetic tree was exported as Newick tree format and the sequences corresponding to the classified OTUs were exported into a fasta file. All exported files from Qiime2 analysis were imported to phyloseq package (McMurdie & Holmes, 2013) on R-CRAN for figure plotting and further analysis. It is important to mention that reads were not rarefied to an even sampling depth (McMurdie & Holmes, 2014). Shannon Entropy was calculated from Fasta sequences of *Streptococcus* and *Escherichia-Shigella* bacterial genera using Oligotyping software (Eren et al., 2014). The threshold was selected as 0.2 (Eren et al., 2014) above which the variation in considered as mutation rather than sequencing error.

## Results

### Sample nature and reads quality

High-throughput sequencing generated a sum of 904,685 reads in both directions (mean reads per sample per direction = 42,407.9 and median reads per sample per direction = 41,645.5). The average read length obtained (Forwards reads ∼280 bp, Reverse reads ∼250 bp). All reads were trimmed to 230 bp after denoising on QIIME 2 (qiime deblur denoise-16S).

### Alpha diversity analysis

**Figure 1:**
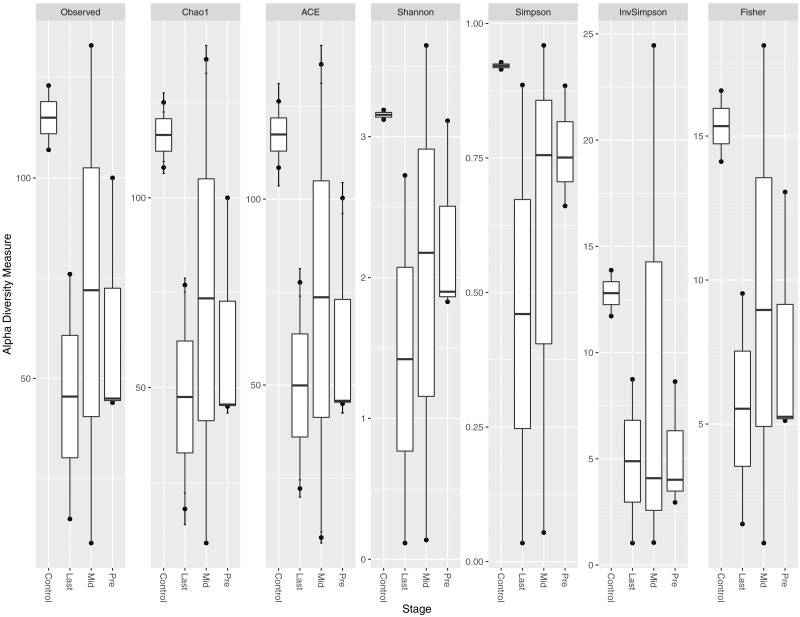
Alpha diversity analysis across different time points. Box plot of different alpha diversity measures (A) Observed OUT, (B) Chao1 index, (C) ACE, (D) Shannon, (E) Simpson, (F) InvSimpson, and (G) Fisher for comparing the samples across the three time points (pre, before exposure to radiation; mid, after 12–15 fractions and Last, after 26–28 fractions of radiation that is equivalent to 50.4 Gy) with controls (never exposed to chemotherapy or radiotherapy before).

Different indices of alpha diversity measures (Observed OTU, Chao1, Shannon, Simpson, InvSimpson, and Fisher) all showed a higher diversity in healthy controls compared to cancer patients in the three-time points collected (Fig. 1). After completing the radiotherapy treatment along with the antibiotics courses (Last), the alpha diversity has generally declined when compared to the mid-point after 12–15 fractions and before radiation. The outliers at each time point were obvious, which could be attributed to the personal variations between the patients, along with the variation due to the set of antibiotics prescribed (antibiotic course duration and antibiotic course timing relative to the radiotherapy and sample collection time).

### Alpha diversity per sample

**Figure 2:**
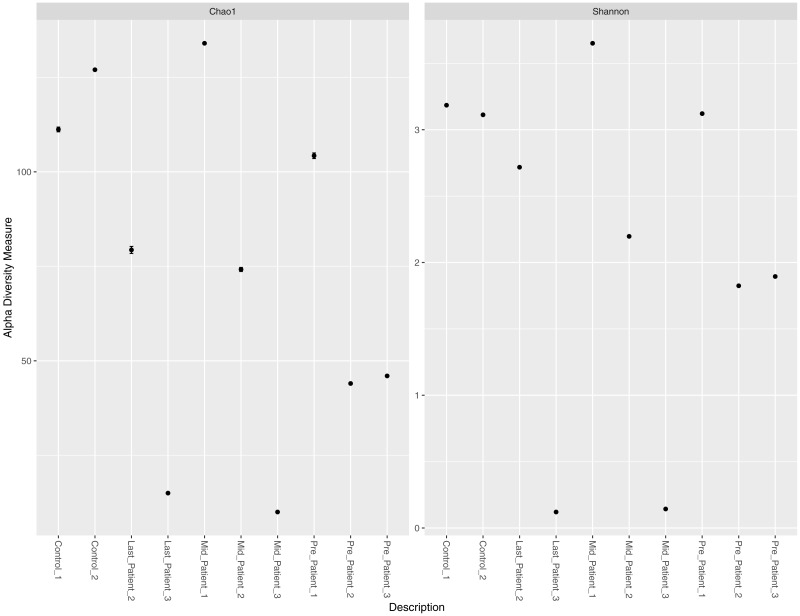
Chao1 and Shannon indices per sample. Chao1 and Shannon indices of alpha diversity per sample. Neither index indicated a direct relationship between exposure to radiation and alpha diversity.

Alpha diversity measures (Chao1 and Shannon) per sample did not indicate a direct relationship between exposure to radiation, intensive use of antibiotics, and abundance of all bacterial species per sample (Fig. 2). Patients-1 and 2 experienced an increase in the alpha diversity after the two aforementioned exposures, unlike patient-3 who experienced a massive drop in alpha diversity. On the other hand, a pattern was inferred between high alpha diversity and response to treatment (Table 2). The low bacterial abundance was associated with a positive response to radiotherapy and vice versa. It is important to mention that the Chao1 index reflect the richness only (number of bacterial species per sample), while Shannon index reflects both richness and evenness (relative abundance of species that make up the richness).

### Variation in the bacterial abundance at different taxonomic levels

**Figure 3:**
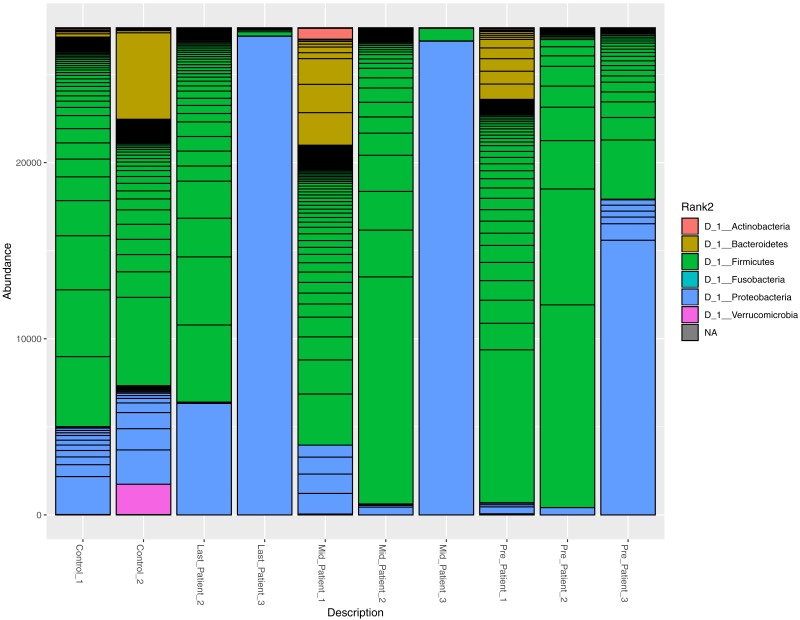
Relative abundance of bacterial species at the phylum level. The abundance of the four major bacterial phyla across different samples. Bar plot showing the relative abundance of the four major phyla in controls and patients. The two healthy controls and patient 1 (in both time points) are quite comparable, this could be due to the lessen exposure of patient 1 to antibiotics. However, the bar plot showed a relative decrease in Firmicutes and increase in Proteobacteria after his exposure to radiation (21.6 Gy).

At the phylum level, the two healthy controls showed normal variation between the four most abundant bacterial phyla (Firmicutes, Bacteroidetes, Proteobacteria and Actinobacteria) (Fig. 3), with domination of Firmicutes and relatively high abundance of Proteobacteria (Odamaki et al., 2016).

After the exposure to different doses of radiation that ranged between (21.6 Gy and 50.4 Gy), along with the antibiotic courses (Table 1), the relative abundance of Firmicutes decreased while the Proteobacteria increased in the three patients. The phylum abundance (Fig. 3) showed disturbance in microbial phyla when compared to controls. However, when comparing each patient to himself (at the different time points collected), it was found that Actinobacteria, Bacteroidetes and Proteobacteria phyla increased after antibiotics and radiation while Firmicutes decreased, which may explain the increase in alpha diversity (Fig. 2).

**Table 2:** Alpha diversity with reflection to the response to radiation.

Sample	Chao1	Shannon	Response to treatment
Control-1	111.2	3.184	NA
Control-2	127	3.11	NA
Pre-patient-1	104.25	3.121	NR
Mid-Patient-1	134	3.651	
Pre-Patient-2	44	1.82	R
Mid-Patient-2	74.167	2.19		
Last-Patient-2	79.333	2.717	
Pre-Patient-3	46	1.894	R
Mid-Patient-3	10	0.143		
Last-patient-3	15	0.12	

**Notes.**

NA Not Applicable R Responded to radiotherapy NR Not responding to radiotherapy

**Figure 4:**
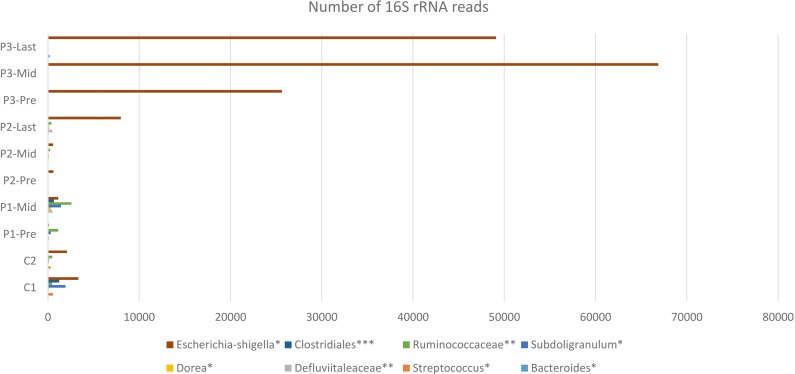
Constant increase of bacterial taxa post Radiotherapy. Post Radiation samples show a massive increase with different taxa represented by the number of 16S rRNA reads. All listed taxa showed increase in their abundance post radiotherapy. However, the highest increase in bacterial abundance was noticed for *Defluviitaleaceae* by 467-fold increase in patient 2. Several genera like *Bacteroides, Streptococcus, Dorea, Subdoligranulum,* and *Escherichia-Shigella* showed increase in their abundance post radiotherapy by maximum fold increase of 192, 5.1, 11, 79, and 13.3, respectively. Family *Defluviitaleaceae* and *Ruminococcaceae* demonstrated maximum fold increase of 467 and 61.3, respectively, while the order *Clostridiales* demonstrated a maximum fold increase of 26.

A higher resolution or an intuition of more specificity to the bacterial taxa was needed. The frequency table at the genus level was obtained for all samples. The search criteria for the specific increasing genera was set as follows: (1) the frequency per patient was higher at each time point (fluctuating taxa were excluded), (2) frequencies per taxa were elevated in the three patients or completely absent in one patient. According to these criteria, eight different genera were identified (Fig. 4), six of which belong to the Firmicutes phylum (that had overall decreased). One belongs to Proteobacteria and the last is a member of Bacteroidetes phylum (Table S1).

### Variation at different nucleotide positions before and after radiotherapy

**Figure 5:**
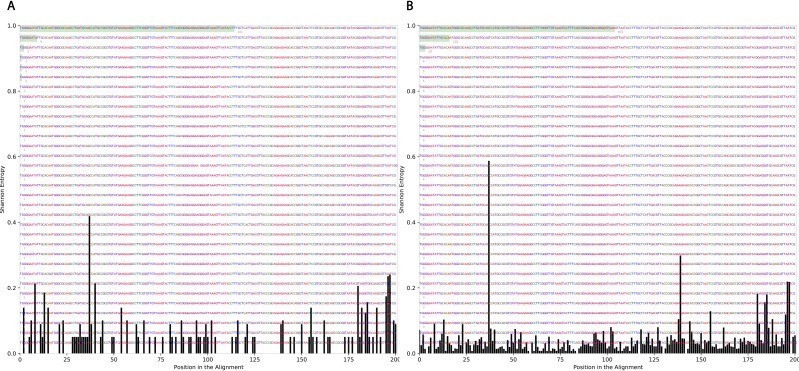
Shannon’s Entropy analysis for fasta reads of *Escherichia-Shigella* before radiotherapy and after radiotherapy using Oligotyping software. Reads alignment of *Escherichia-Shigella* genus before radiation showed minimal variation, only single variation increased above the threshold (0.2 Shannon’s entropy) between base 25 and 50. After radiation the entropy at the same position increased to ∼0.6 while three other positions experienced higher variation above the threshold (between base 125 and 150, between 175 and 200).

**Figure 6:**
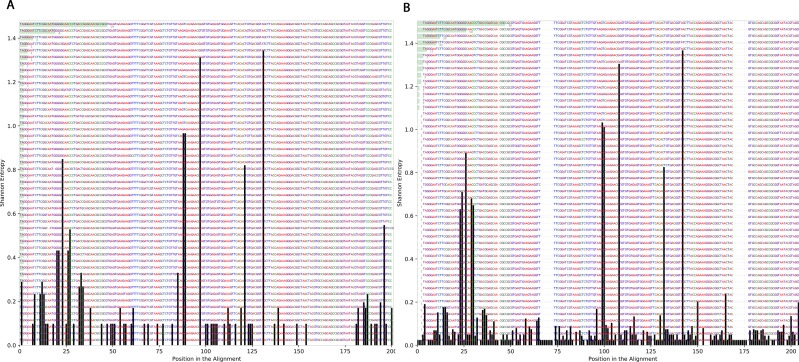
Shannon’s Entropy analysis for fasta reads of *Streptococcus* before radiotherapy and after radiotherapy using Oligotyping software. *Streptococcus* genus reads before and after radiotherapy. Although the increase in Shannon’s entropy post radiotherapy is minimal, deletion at two positions are clear (between 50 and 70/between 150 and 175).

To determine the effect of exposure to intensive radiation on the variation in bacterial DNA sequences, Shannon entropy was determined. The reads used in the analysis were trimmed to 200 bases to exclude low quality base calling. From the bacterial genera identified (Table S1), two were randomly selected to determine Shannon entropy at a single nucleotide position (comparing before radiation to after radiation at the same nucleotide position). The entitled analysis was performed on *Streptococcus* and *Escherichia-Shigella.* Shannon entropy increased (Figs. 5 and 6) at definite nucleotide positions in both genera and post exposure to radiation. Values below 0.2 were considered as sequencing errors and accordingly excluded. *Escherichia-Shigella,* gap insertions were clear (Fig. 5) to reach the best sequence alignment, which indicates a high probability of single point mutations in the bacterial DNA post radiotherapy. It is important to note that the Shannon entropy was performed only on a definite sequence length (approx. 200 bp from V3–V4 region of the 16S rRNA of the bacterial genome).

### Beta diversity analysis

**Figure 7:**
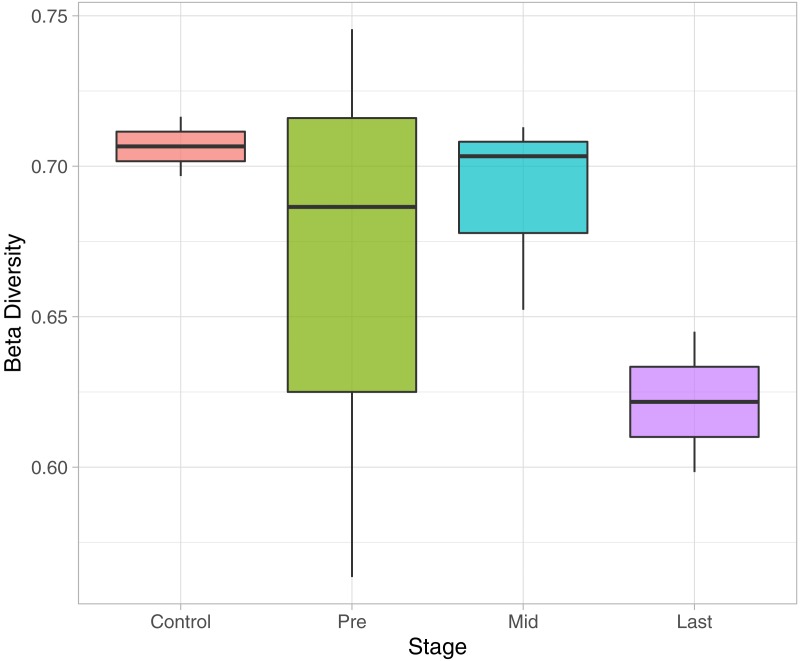
Beta Diversity analysis based on Bray-Curtis metrics. The Box-plot employs the variation within each group. The groups were classified by stages with referral to the extent of exposure to radiation. Control, never exposed to either chemotherapy or radiotherapy; pre, undergoing chemotherapy but are not exposed to radiation yet; Mid, underwent 12–16 radiotherapy sessions; and Last, underwent 26–28 radiotherapy sessions.

To determine the effect of increasing the exposure of radiation on the bacterial diversity, beta diversity analysis was performed based on Bray-Curtis dissimilarity metric. Samples were grouped according to the stage of sample collection (Fig. 7). The bacterial diversity appears to decline with the stage (by increasing the exposure to radiation). The control samples share a high extent of similarity in terms of the bacterial taxa presence in each sample, while the pre samples had the highest recorded level of variation among the bacterial taxa per sample. Finally, for the mid and last sample groups, both the beta diversity, across the groups and across the samples in the same group, decreased as the exposure to radiation increased.

## Discussion

The 16S rRNA sequencing profile in pediatric cancer patients, suffering from rhabdomyosarcoma near the pelvic region before and after exposure to radiotherapy, was described. Comparing the alpha diversity of patients before radiotherapy to healthy controls showed an expected variation due to prior treatment with chemotherapy and antibiotics, as the healthy controls had higher alpha diversity. While alpha diversity per individual before and after treatment did not follow a constant pattern post radiation, some patients had experienced an increase except of one who experienced a decrease in alpha diversity post exposure to radiation. The discrepancy could be linked to treatment response, since the responders had low bacterial abundance while the non-responder had higher bacterial abundance. This observation comes in agreement with Crawford and colleagues who described the effect of radiation on germ free mice that were found to be resistant to lethal doses of ionizing radiation (Crawford & Gordon, 2005).

It was also consistent across all samples to find that the members of Firmicutes phylum are highly affected by radiation that was also described by Young-Do Nam and colleagues who reported similar findings in gynecological cancer patients exposed to radiotherapy (Nam et al., 2013). The decrease in Firmicutes was associated with Proteobacteria increase in three patients. This pattern agreed with previous results of Wang and colleagues (Wang et al., 2015). The Shannon entropy analysis shed the light on the possibility of DNA mutations in the bacterial taxa that were found to be resistant to radiotherapy and were constantly increasing across all samples. The choice of 16S rRNA encoding gene for mutation analysis could be controversial due to sequence variability in both variable and the conserved regions (Větrovský & Baldrian, 2013; Yang, Wang & Qian, 2016; Martinez-Porchas et al., 2017). Oligotyping software also provides values for Shannon’s entropy, through which the changes could be judged and referred to sequencing error (values equal to or below 0.2 are excluded) or real mutations/changes due to external factors being tested. Accordingly, this pilot survey can be followed up with further mutational analyses across different bacterial genes post radiation to study the potential impact of radiation on acquiring the bacteria resistance to antibiotic.

The incidence of pediatric cancers (<18 years) was reported to occur in a rate of 140.6 per million child in the age group 0–14 years (between years: 2000–2010) (Steliarova-Foucher et al., 2017). The treatment protocols usually combine chemoradiotherapy with prophylactic antibiotics to suppress any possible infection because the patient’s immune system is highly compromised. The gut microbiome in children starts to develop untill reaching the adults composition. Therefore, it is crucial to detect any imbalance occurring at this critical stage due to cancer or treatment. Moreover, several studies linked the state of microbiome to the response of treatment (Iida et al., 2013; Alexander et al., 2017; Gerassy-Vainberg et al., 2018; Gopalakrishnan et al., 2018). Thus, such profiling before and after treatment is highly needed to aid the physicians in determining the treatment plan for each patient according to his/her microbial diversity.

## Conclusions

The microbial profile was described in the gut of pediatric cancer patients in time course with exposure to radiation near the pelvic region. In addition, a relationship between the microbial diversity and response to treatment was inferred, in which the increase in alpha diversity was related to a non-responsive radiotherapy treatment. Such a relationship could be useful in the prediction of response to treatment or the enhancement of treatment by microbial remolding. Moreover, a close insight to the bacterial 16S rRNA sequence via multiple sequence alignment to determine Shannon’s entropy was described, before and after 50.4 Gy of radiation directed to the pelvic region. Several indels were pinpointed in the 16S rRNA sequences after the radiation course, which indicates a high probability of finding other mutations in the bacterial genome post exposure to radiation that might be detrimental. Finally, a decline in beta diversity was recorded along with the increasing in exposure to radiation. Although this study had limitations in the number of participants and sampling points due to the nature of cancer and patients’ age, it gives an initial insight for the possible mutations occurring in the bacterial DNA post cancer treatment and the link between the microbial diversity and the response to treatment. The study is a start to offer a different angle for personalized treatment progress for pediatric cancer patients, based on the microbial profile rather than following a constant roadmap for the treatment protocol.

##  Supplemental Information

10.7717/peerj.7683/supp-1 Table S1 Full taxonomic classification of the constantly increasing genera

10.7717/peerj.7683/supp-2 Supplemental Information 2 Scripts used in sequence analysis

10.7717/peerj.7683/supp-3 Supplemental Information 3 16S rRNA reads of cancer patient 1 before starting radiotherapy sessions Male at age of 7, taking a sample from patient 1 (P1) at time zero, 12 days before exposed to his first radiation dose.

10.7717/peerj.7683/supp-4 Supplemental Information 4 16S rRNA reads of cancer patient 1 after 12 days from starting radiotherapy sessions Radiotherapy fraction 21.6 Gy
Male at age of 7
First dose after 12 days from taking a sample from patient 1 (P1)

10.7717/peerj.7683/supp-5 Supplemental Information 5 16S rRNA reads of cancer patient 2 before starting radiotherapy sessions Male at age of 7, taking a sample from patient 2 (P2) at time zero, 14 days before exposed to his first radiation dose.

10.7717/peerj.7683/supp-6 Supplemental Information 6 16S rRNA reads of cancer patient 2 after 14 days from starting radiotherapy sessions Radiotherapy fraction 25.2 Gy, Male at age of 7, First dose after 14 days from taking a sample from patient 2 (P2)

10.7717/peerj.7683/supp-7 Supplemental Information 7 16S rRNA reads of cancer patient 2 after 26 days from starting radiotherapy sessions Radiotherapy fraction 46.8 Gy, Male at age of 7, second dose after 12 days from the first dose from patient 2 (P2)

10.7717/peerj.7683/supp-8 Supplemental Information 8 16S rRNA reads of cancer patient 3 before starting radiotherapy sessions Male at age of 4, taking a sample from patient 3 (P3) at time zero, 16 days before exposed to his first radiation dose.

10.7717/peerj.7683/supp-9 Supplemental Information 9 16S rRNA reads of cancer patient 3 after 16 days from starting radiotherapy sessions Radiotherapy fraction 28.8 Gy, Male at age of 4, First dose after 16 days from taking a sample from patient 3 (P3)

10.7717/peerj.7683/supp-10 Supplemental Information 10 16S rRNA reads of cancer patient 3 after 27 days from starting radiotherapy sessions Radiotherapy fraction 48.6 Gy, Male at age of 4, second dose after 11 days from the first dose from patient 3 (P3)

10.7717/peerj.7683/supp-11 Supplemental Information 11 16S rRNA reads of healthy individual 1 Male at age of 3.6, taking a sample from healthy kid 1 as control 1 (C1).

10.7717/peerj.7683/supp-12 Supplemental Information 12 16S rRNA reads of healthy individual 2 Male at age of 4.5, taking a sample from healthy kid 2 as control 2 (C2).

## Additional Information and Declarations

### Data Availability

The following information was supplied regarding data availability: Sequences are available at NCBI, BioProject: PRJNA545788.
