# Supplementary material for: Effect of radiotherapy on the gut microbiome in pediatric cancer patients: a pilot study
Source: PeerJ. 2019 Sep 23;7:e7683. doi: 10.7717/peerj.7683 (PMC6761921; doi:10.7717/peerj.7683)
Supplement: Table S1 [file peerj-07-7683-s001.docx]

**Table S1: Full taxonomic classification of the constantly increasing genera**

| **Kingdom** | **Phylum** | **Class** | **Order** | **Family** | **Genus** |
| --- | --- | --- | --- | --- | --- |
| Bacteria | Bacteroidetes | Bacteroidia | Bacteroidales | Bacteroidaceae | *Bacteroides* |
| Bacteria | Firmicutes | Bacilli | Lactobacillales | Streptococcaceae | *Streptococcus* |
| Bacteria | Firmicutes | Clostridia | Clostridiales | Defluviitaleaceae | uncultured |
| Bacteria | Firmicutes | Clostridia | Clostridiales | Lachnospiraceae | *Dorea* |
| Bacteria | Firmicutes | Clostridia | Clostridiales | Ruminococcaceae | *Subdoligranulum* |
| Bacteria | Firmicutes | Clostridia | Clostridiales | Ruminococcaceae | NA |
| Bacteria | Firmicutes | Clostridia | Clostridiales | NA | NA |
| Bacteria | Proteobacteria | Gammaproteo bacteria | Enterobacteriales | Enterobacteriaceae | *Escherichia- Shigella* |
